# Comparative Analysis of Two Gene-Targeting Approaches Challenges the Tumor-Suppressive Role of the Protein Kinase MK5/PRAK

**DOI:** 10.1371/journal.pone.0136138

**Published:** 2015-08-21

**Authors:** Natalia Ronkina, Claus Johansen, Lisa Bohlmann, Juri Lafera, Manoj B. Menon, Christopher Tiedje, Kathrin Laaß, Benjamin E. Turk, Lars Iversen, Alexey Kotlyarov, Matthias Gaestel

**Affiliations:** 1 Department of Biochemistry, Hannover Medical School, Hannover, Germany; 2 Department of Dermatology, Aarhus University Hospital, Aarhus C, Denmark; 3 Department of Pharmacology, Yale University School of Medicine, New Haven, United States of America; National Cheng Kung University, TAIWAN

## Abstract

MK5 (MAPK-activated protein kinase 5) or PRAK (p38-regulated and -activated kinase) are alternative names for a serine/threonine protein kinase downstream to ERK3/4 and p38 MAPK. A previous gene targeting approach for MK5/PRAK (termed here MK5/PRAK-Δex8) revealed a seemingly tumor-suppressive role of MK5/PRAK in DMBA-induced one step skin carcinogenesis and Ras-induced transformation. Here we demonstrate that an alternative targeting strategy of MK5/PRAK (termed MK5/PRAK-Δex6) increased neither tumor incidence in the one step skin carcinogenesis model, nor Ras-induced transformation in primary cells. Interestingly, due to the targeting strategies and exon skipping both knockouts do not completely abolish the generation of MK5/PRAK protein, but express MK5/PRAK deletion mutants with different biochemical properties depending on the exon targeted: Targeting of exon 6 leads to expression of an unstable cytoplasmic catalytically inactive MK5/PRAK-Δex6 mutant while targeting of exon 8 results in a more stable nuclear MK5/PRAK-Δex8 mutant with residual catalytic activity. The different properties of the MK5/PRAK deletion mutants could be responsible for the observed discrepancy between the knockout strains and challenge the role of MK5/PRAK in p53-dependent tumor suppression. Further MK5/PRAK knockout and knock-in mouse strains will be necessary to assign a physiological function to MK5/PRAK in this model organism.

## Introduction

MAPK-activated protein kinase 5 (MAPKAPK5, MK5)/p38-regulated and -activated kinase (PRAK) is a distant relative of the MAPKAPKs MK2 and MK3, which are activated by p38 MAPK upon stress stimulation. The pathways regulating expression and activity of MK5/PRAK *in vivo* are not completely understood and may comprise both the conventional MAPK p38alpha as well as atypical MAPKs, such as ERK3 and ERK4 (reviewed in [1,2]), and its acetylation by Tip60 [3]. Downstream to MK5, various transcription activators, such as p53 and members of the forkhead family FoxO, and the Ras homologue enriched in brain Rheb have recently been identified [4–7]. Remarkably, the ERK3/4-MK5-pathway could possess both pro- and anti-oncogenic properties (reviewed in [8]).

The physiological role of MK5/PRAK has mainly been analyzed using two independently generated mouse models: The “Δex6 mice”, in which exon 6 of MK5/PRAK is replaced by a neomycin cassette and which are kept in the 129xC57BL/6 background to increase viability and fertility [9], and the “Δex8 mice” where exon 8 was replaced [6]. Our initial characterization of the Δex6 mouse showed a loss of basal enzymatic activity of MK5/PRAK, which could not be stimulated upon classical p38 MAPK-activating stresses in wild type cells, but indicated no involvement of MK5/PRAK in innate immunity and inflammation [9], where the p38 MAPK-activated protein kinases MK2 and MK3 are of essential importance [10]. More interestingly, analysis of the Δex8 mouse demonstrated a profound p53-dependent role of MK5/PRAK in tumor suppression [6]. This role was initially demonstrated by (i) increased tumor formation in Δex8 mice in the one-step DMBA skin carcinogenesis model, (ii) decreased p21WAF expression in Δex8 H-Ras-G12V-transformed MEFs, (iii) increased colony formation of H-Ras-G12V-transformed Δex8 primary cells, and (iv) finally explained by phosphorylation of p53 by PRAK at serine residue S37 [6]. Subsequently, using Δex8 mice in the two-step DMBA skin carcinogenesis model it was then proved that in addition to the early tumor-suppressing function of MK5/PRAK a late tumor-promoting function of the p38-MK5/PRAK pathway exists, where MK5/PRAK acts as an angiogenic and cell migration stimulating host factor [11]. In addition, using the Δex8 mice in a mouse model harboring the oncogenic ras allele N-Ras-G12D specifically expressed in hematopoietic cells, enhanced hematopoietic tumorigenesis was observed which supported the notion that MK5/PRAK functions as a tumor suppressor in multiple types of cancers [12].

Here, we analyzed the apparent tumor-suppressive function of MK5/PRAK using the Δex6 mice generated by us previously [9]. We repeated key experiments performed by Sun and colleagues [6] and showed no influence of MK5/PRAK targeting on tumorigenity in the one step DMBA skin carcinogenesis model or in Ras-induced transformation experiments. By comprehensive investigation of the differences between both MK5/PRAK knockout strains we detected MK5/PRAK truncated protein variants in both Δex8 and Δex6 cells. Although, we cannot exclude other reasons, the presence of MK5/PRAK artificially truncated remnants distinctive in their stability, localization and catalytic activity is one possible explanation for the observed difference in the phenotype of two MK5/PRAK knockout strains. Regardless of the exact molecular reason, our results challenge the role of MK5/PRAK in tumorigenesis.

## Results

### Different phenotypes of Δex6 and Δex8 mice

**Fig 1:**
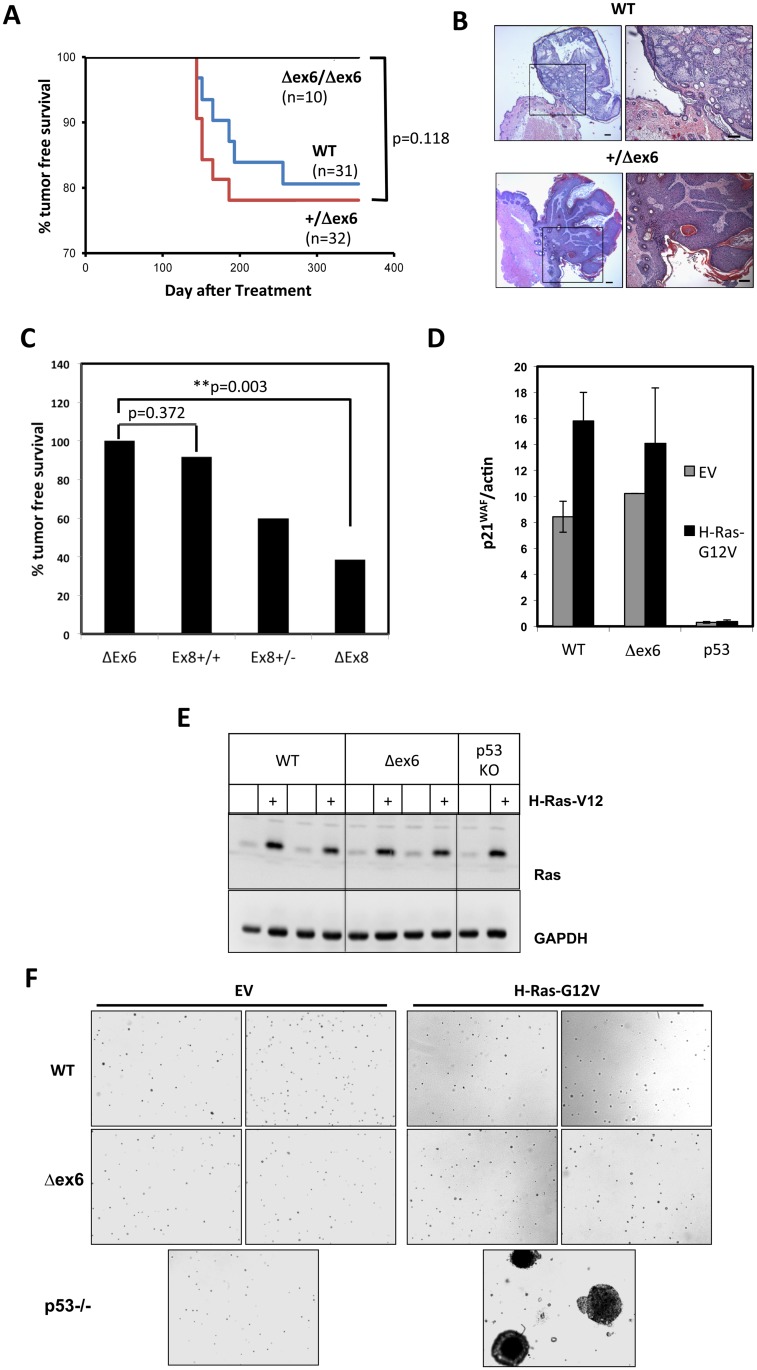
Targeting exon 6 of MK5/PRAK leads to an opposite phenotype than targeting exon 8. A) Tumor free survival of WT, MK5/PRAK +/Δex6 and MK5/PRAK Δex6/Δex6 mice in the one-step DMBA skin tumor model. B) Hematoxylin/eosin stain of similar papillomas formed in WT and MK5/PRAK +/Δex6 mice in the one-step DMBA skin tumor model. C) Comparison of the survival of MK5/PRAK Δex6, exon8+/+ (ex8+/+), exon8+/- (ex8+/-) and Δex8 mice in the one-step skin tumor model. The data for MK5/PRAK ex8+/+, ex8+/- and Δex8 tumor-free survival were taken from [6,9] and significance of the differences was estimated from the measured Δex6 and the compiled ex8+/+ and Δex8 data from the graph of Fig 1A of [6,9] by log-rank test. D) No reduction in p21 mRNA expression in primary H-Ras-G12V-transduced Δex6 MEFs. E) Wild type, Δex6 MEFs and as positive control primary p53-/- MEFs were transduced with H-Ras-G12V or empty vector (EV) and F) Colony formation of the transduced MEFs in soft agar was analyzed by visual inspection of two independently transduced cell populations. Increased colony formation could only be detected for Ras-transduced p53-deficient cells. Colony formation of H-Ras-G12V-transduced Δex6 MEFs in soft agar is similar to the wild type control and to the EV transduced MEFs. The data are representative for three independent experiments each performed in duplicates.

In previous experiments the one step DMBA treatment of mice resulted in superficial papillomas in 8% of wild type animals (1of 12) after about 300 days, whereas 54% (7 of 13) of Δex8/Δex8 mice and 40% (12 of 30) of Δex8/+ mice developed papillomas, indicating an important role of MK5/PRAK in tumor suppression [6,13]. To verify the tumor suppressive properties of MK5/PRAK, we decided to analyze also Δex6 mice in the one-step DMBA model. Unexpectedly, Δex6 mice did not show increased skin tumor formation in this model, which led to superficial papillomas in about 20% of wild type animals after 300 days (Fig 1A and 1B). Tumor free survival of homozygous Δex6 mice was not significantly higher (p-value 0.170,) than that of wild type animals (Fig 1A) but differed significantly from the tumor free survival rate described for heterozygous and homozygous Δex8 mice in the same model (S1 Fig) [6].

The cyclin-dependent kinase inhibitor p21^WAF1^ is a key marker molecule of tumor suppression and ras-induced senescence [14]. In MEFs derived from Δex8 mice, H-Ras-G12V-induced p21^WAF^ expression was significantly decreased and it was postulated that the presence of MK5/PRAK stimulates the activity of p53 by phosphorylation of serine 37 to increase p21^WAF1^ expression [6]. In contrast, in primary H-Ras-G12V-transduced Δex6 MEFs we could not detect any reduction of p21^WAF1^ transcript level while p53-deficient primary MEFs, as positive control, show almost complete abrogation of p21WAF expression (Fig 1D) indicating another profound difference in the phenotypes of Δex6 and Δex8 mice. In addition, using the highly specific p38 MAPK inhibitor BIRB796 at 1μM we found that the strong phosphorylation of p53 at serines S15, S37, and S46 in DNA-damaging agent doxorubicin- and UV-treated MCF-7 cells does not depend on p38 MAPK, although p38 MAPK is strongly activated by these stimuli as seen by phosphorylation of the downstream target Hsp27 (Panel A in S2 Fig). This is in apparent contrast to previous results demonstrating effects of the p38 MAPK inhibitors SB203580 and SB202190 at 20μM and 40μM on UV-induced p53 phosphorylation at S15 and S37 in MCF-7 cells [15]. However, the high inhibitor concentrations used in the previous study could result in cross-inhibition of other protein kinases and were not suited to demonstrate p38 MAPK-specific effects [16,17]. Although we demonstrate that p53 phosphorylation at S37 upon genotoxic-stress and UV-irradiation is not p38 MAPK dependent, we can not completely exclude that oncogenic H-Ras-induced phosphorylation of p53 at S37 in human fibroblasts could proceed via p38 MAPK and MK5/PRAK by an mechanism which requires additional and oncogenic H-Ras-specific signals for the activation of MK5/PRAK by catalytically-active p38 MAPK. In a p53 reporter gene assay in MCF-7 cells, co-expression of p38 MAPK and MK5/PRAK had only minor effect on the transcriptional activity of p53 and this effect was independent of catalytic activity of MK5/PRAK, since co-expression of the MK5/PRAK catalytic-dead mutant (MK5-T182A), which displays significantly lower basal activity and cannot be further activated by p38 MAPK [18–21], has comparable effect on reporter activity as the wild type kinase (Panel B in S2 Fig). Here, we can also not completely exclude that under overexpression conditions, the basal kinase activity of MK5 or MK5-T182A is sufficient to phosphorylate and activate the endogenous p53 protein in MCF7. Interestingly, co-expression of p38 MAPK together with MK5 in HEK293 cells strongly activates MK5 catalytic activity towards Hsp27 (see also below), but co-expression of p38 MAPK does not further enhance the ability of MK5 to induce p53-dependent reporter activity in MCF7 cells (Panel B in S2 Fig), indicating that increase in MK5 catalytic activity does not result in increased activity of endogenous p53. In addition, the phosphorylation site consensus motif of MK5/PRAK displays a mandatory arginine (R) in -3 position, which can not be found in p53 at position 34 (Panel C in S2 Fig), making the direct phosphorylation of p53 at S37 by MK5/PRAK again rather unlikely.

For Δex8 primary MEFs a promotion of H-Ras-G12 V induced transformation comparable to that of p53-deficiency was demonstrated by increased colony formation in soft agar [6]. Therefore, we analyzed colony formation of H-Ras-G12V transduced primary Δex6-MEFs and, as positive control, of H-Ras-G12V transduced p53-deficient MEFs (Fig 1E) using the identical expression vector [22] in soft agar (Fig 1F). While H-Ras-G12V transduced p53-deficient MEFs displayed increased colony growth in soft agar, no difference between H-Ras-G12V transduced wild type and Δex6-MEFs and empty vector transduced cells in soft agar colony formation was detected (Fig 1F). Collectively these data show that Δex8 and Δex6 mice and cells derived thereof display clear differences in key features of oncogenic phenotypes.

### Detection of MK5/PRAK mutant mRNA

**Fig 2:**
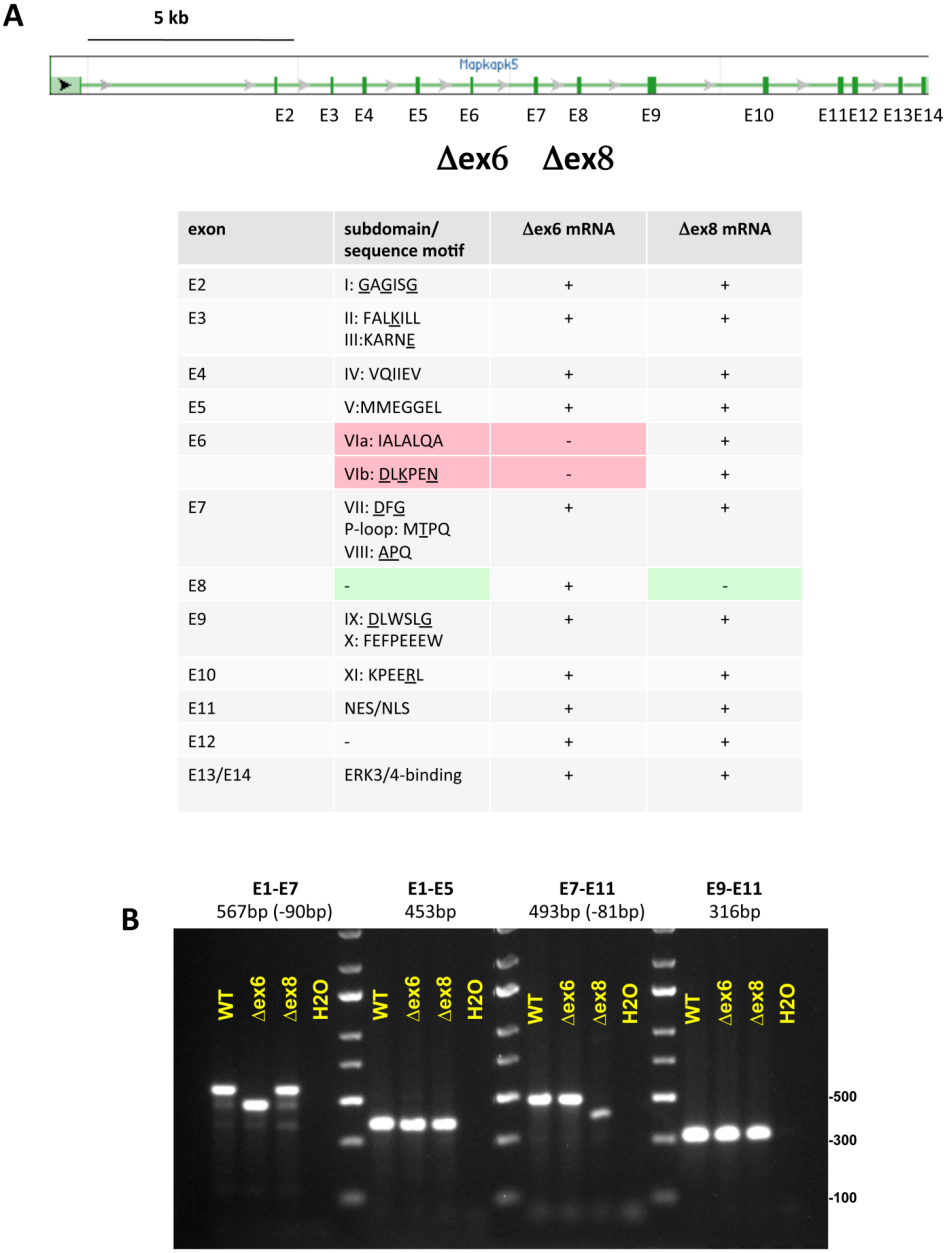
Detection of mutant MK5/PRAK mRNAs in Δex6 and Δex8 MEFs. A) MK5/PRAK gene structure and assignment of the conserved protein kinase subdomains, the overlapping nuclear export (NES)/ nuclear localization signal (NLS) and the ERK3/4-binding site [6,37] to the different exons. B) Detection of MK5/PRAK mRNA fragments from Δex6 and Δex8 MEFs by exon specific PCR.

Since the differences described above may result from the different targeting strategies, we compared the targeted exons and the resulting MK5/PRAK transcripts. Exon 6 encodes the essential protein kinase catalytic subdomains VIa and VIb of MK5/PRAK (Fig 2A). Its targeting strategy involved the replacement of Exon 6 together with some intronic regions (800bp) by a neomycin cassette [9]. This results in the expression of a truncated mRNA which could code for a protein kinase without catalytic activity. Indeed, a weak band representing a truncated MK5/PRAK protein completely devoid of catalytic activity was detected in some tissues and cells of Δex6 mice in our previous study [9]. In the other mouse strain, a major part of exon 8, which codes for a sequence C-terminal to catalytic subdomain VIII of MK5/PRAK that is dispensable for its catalytic activity (Fig 2A), was replaced by a targeting cassette, where a self-cleaving ribozyme sequence was included in opposite orientation with the neomycin cassette to destroy possible truncated or fusion-mRNAs [6]. However, removal of this alternate exon, containing the targeting cassette and 5’ sequences of Exon 8, by exon skipping might also delete the self-cleaving sequence and stabilize the mRNA.

We analyzed MK5/PRAK transcripts of Δex6 and Δex8 MEFs by RT-PCR. As expected, we were able to amplify MK5/PRAK mRNA fragments spanning exons 1–5, 7–11, 9–11 as well as 1–7 lacking the 90 bases of exon 6 from Δex6 MEFs (Fig 2B). In contrast to Sun and colleagues who could not detect any mRNA or protein traces of MK5/PRAK in the Δex8 knockout [6], we were able to detect a considerable amount of MK5/PRAK mRNA fragments from Δex8 MEFs. Here, regions spanning exons 1–7, 1–5, 9–11 and most importantly 7–11 lacking the 81 bases of exon 8 could be detected (Fig 2B). This indicates that exon skipping took place for the exon8-targeted gene and that the self-cleaving ribozyme sequence introduced in the Δex8 knockout cassette was not efficient before splicing. The targeted genotype of the MEFs analyzed above was confirmed by PCR using genomic DNA isolated from these cells and cassette-specific primers (S3 Fig).

### Detection of residual truncated MK5/PRAK proteins with different stability, subcellular localization and catalytic activity

**Fig 3:**
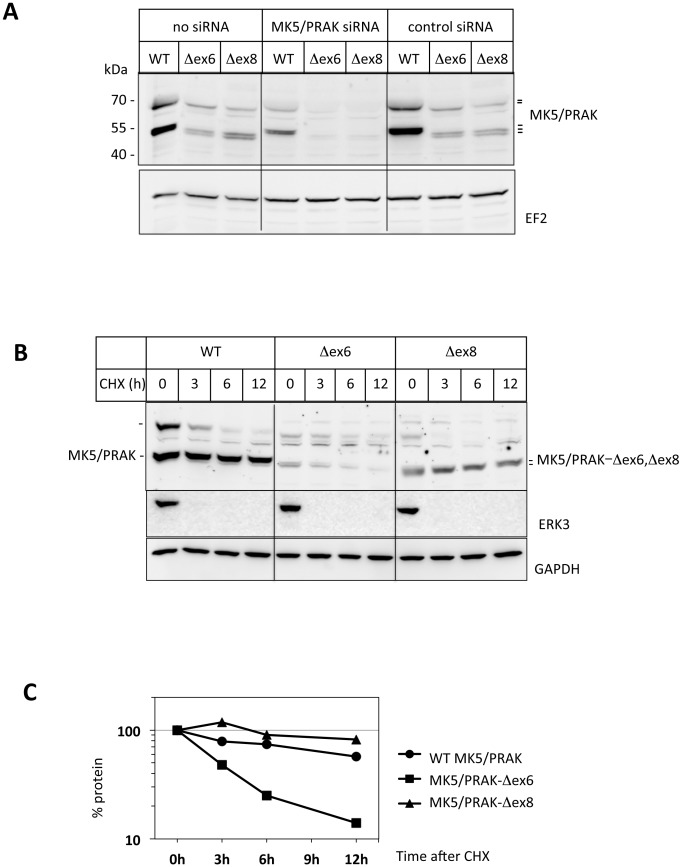
Detection of protein mutants of MK5/PRAK and analysis of their stability. A) Detection of MK5/PRAK proteins in lysates from primary Δex6 and Δex8 MEFs. Specificity of the signal is demonstrated by MK5/PRAK siRNA treatment. Loading control was performed by Western blot of elongation factor 2 (EF2). According to the molecular weight marker shown left, the three MK5/PRAK specific bands, which are reduced by MK5/PRAK siRNA treatment, migrate with apparent molecular masses of about 66 kDa, 52 kDa and 50 kDa (double band). The 52 kDa band fits best to the calculated mass of full length MK5/PRAK (1–473) of 54,152 Da. The 50 kDa band could represent the 471 amino acid splice variant lacking “GK” [30], since exchange or loss of one or a few charged amino acids (“K”) often changes migration in SDS-PAGE for a few kDa. As also described for endogenous mouse MK2/MAPKAPK2, where an additional band of about 56 kDa is detected in addition to the band corresponding to the calculated mass of 44,050 Da for mouse MK2 [38], there is a band detected for endogenous (but not overexpressed) mouse MK5/PRAK which migrates significantly slower than expected from the calculated molecular mass (66 kDa instead 52 KDa). As for MK2, we do not know the reason for this slower migrating band of MK5/PRAK so far. B, C) Stability of endogenous MK5/PRAK proteins was monitored by Western blot (B) and chemiluminescence quantification (C) at different times after cycloheximide (CHX) treatment of MEFs. The program ImageJ 1.38x (NIH- http://rsb.info.nih.gov/ij/) was used for chemiluminescence quantification.

To test whether the mutant mRNA in the targeted cells are translated to protein, we analyzed lysates of Δex6 and Δex8 MEFs by Western blot using anti-MK5 antibodies. As control for the specificity of the signals we resorted to siRNA-mediated MK5/PRAK knockdown. In wild type MEFs, MK5/PRAK is represented by three distinctly migrating bands. Both Δex6 and Δex8 MEFs display expression of slightly faster migrating MK5/PRAK bands, which are specifically reduced by siRNA-mediated MK5/PRAK knockdown (Fig 3A). These bands represent most probably the mutant MK5/PRAK proteins lacking amino acids 131–160 corresponding to exon 6 (MK5/PRAK-ΔEx6) and amino acids 194–220 corresponding to exon 8 *(*MK5/PRAK-ΔEx8), respectively. Hence, mutant MK5/PRAK proteins are expressed in cells of both mouse strains.

To analyze whether the truncated proteins MK5/PRAK-Δex6 and MK5/PRAK-Δex8 differ in their biochemical properties, we first monitored their stability in MEFs after inhibition of protein synthesis by cycloheximide. MK5/PRAK-Δex6 is less stable displaying an estimated half-life of about 3 h, while MK5/PRAK-Δex8 and wild type MK5/PRAK are rather stable proteins with half-life greater than 12 h (Fig 3B and 3C). The low stability of MK5/PRAK-Δex6 is probably also the reason for the reduced expression of this protein compared to WT and MK5/PRAK-ΔEx8 (Fig 3A).

**Fig 4:**
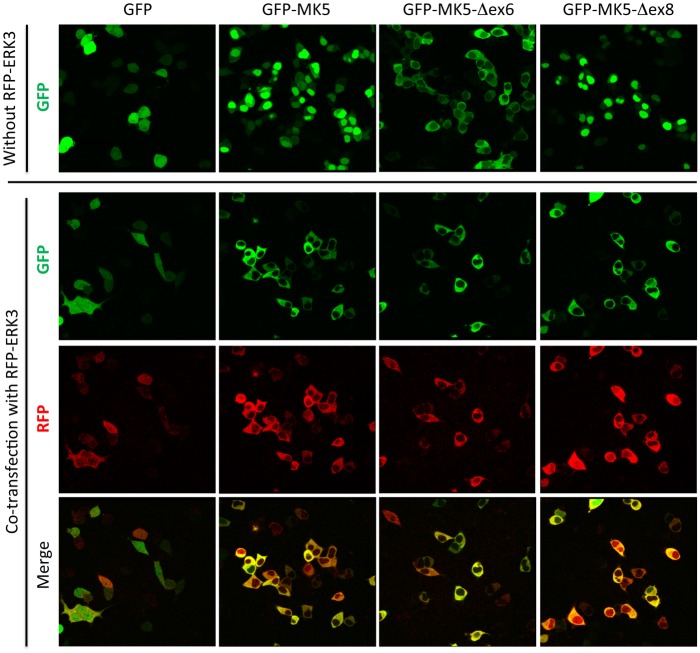
Altered subcellular localization of GFP- MK5-Δex6, but not of GFP- MK5-Δex8, in HEK293 cells and effect of co-expression of ERK3. GFP and GFP-fusions of MK5/PRAK and its mutants were expressed in HEK293 cells in the absence or presence of co-expressed red fluorescent protein (RFP)-fused ERK3 and subcellular localization was followed by confocal fluorescence microscopy.

Next, subcellular localization of MK5/PRAK-Δex6 and MK5/PRAK-Δex8 was determined by cloning the corresponding cDNAs into GFP-fusion expression vectors and transfecting HEK293 cells. While GFP-MK5/PRAK-Δex8 displayed nuclear localization similar to the wild type protein, GFP-MK5/PRAK-Δex6 was excluded from the nucleus and mainly localized in the cytosolic compartment (Fig 4 upper panel and S4 Fig). Interestingly, co-expression of ERK3 renders the nuclear localization of WT and Δex8 to a cytoplasmic one, similar to Δex6 (Fig 4 lower panel).

**Fig 5:**
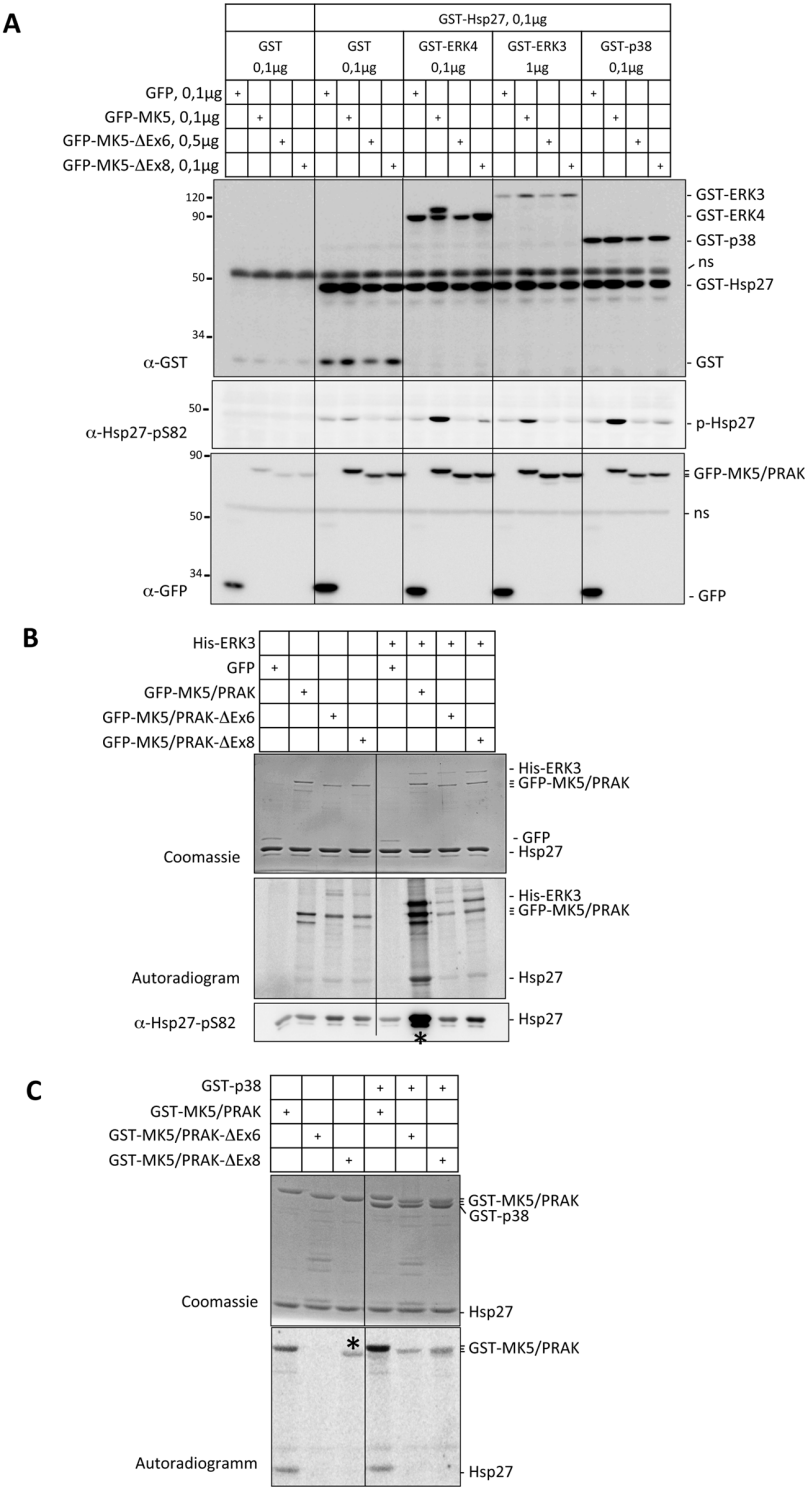
Kinase activity of wild type MK5/PRAK, MK5/PRAK-Δex6 and -Δex8. A) HEK293 cells were transfected with the constructs indicated. The total amount of DNA per well was kept constant (1,6μg DNA per well in 12-well plate) by adding pcDNA-flag expression vector. 24 h post transfection cells were lysed in the plates with 1x Laemmli buffer. Lysates were analyzed by Western blotting using the indicated antibodies. A significant increase in phosphorylation of the substrate Hsp27 was only detected for WT MK5/PRAK in the presence of overexpressed p38 MAPK, ERK3 or ERK4. B) HEK293 cells were transfected with the expression constructs indicated. 24 h post transfection the cells were lysed and GFP-tagged MK5/PRAK or its exon 6 (Δex6) or exon 8 (Δex8) deletion mutants were immuno-precipitated by GFP nanobodies coupled to M270 epoxy beads. The beads were used in a kinase reaction with recombinant Hsp27 as a substrate. The reaction mixture was resolved by SDS-PAGE and Hsp27 phosphorylating activity was detected by phospho-imaging (autoradiogram) and Western blot against pS82-Hsp27. Increased Hsp27-kinase activity is only detected for WT MK5/PRAK (asterisk). C) In vitro MK5/PRAK kinase assay with Hsp27 as substrates. GST–p38 was used to activate MK5/PRAK in vitro. While Hsp27-kinase activity is only detected for WT MK5/PRAK, auto-phosphorylating activity in the absence of p38 MAPK (left panel) is detected for both WT MK5/PRAK and Δex8 (asterisk).

We then determined the catalytic activity of GFP-tagged MK5/PRAK, MK5/PRAK-Δex6 and MK5/PRAK-Δex8 expressed in HEK293 cells alone or in combination with expression of GST-p38, -ERK3 and -ERK4 against the *bona fide* substrate Hsp27 (GST-Hsp27). Significantly increased phosphorylation of GST-Hsp27 at its major site S82 was only detected for the wild type MK5/PRAK protein in the presence of p38, ERK3 and ERK4 (Fig 5A), indicating absence of catalytic activity against Hsp27 in both MK5/PRAK-ΔEx6 and MK5/PRAK-ΔEx8 protein. We also analyzed *in vitro* phosphorylation of recombinant Hsp27 by GFP-fusions of MK5/PRAK, MK5/PRAK-Δex6 and MK5/PRAK-Δex8 immuno-precipitated from HEK293 cells expressing these constructs alone or in combination with His-ERK3. In GST-pull-down experiments all MK5/PRAK proteins demonstrated similarly strong degrees of interaction with His-ERK3 (Fig 5B—Coomassie stain—and S5 Fig). However, increased phosphorylation of the *in vitro*-substrate Hsp27 at its major site S82 was only detected for the wild type MK5/PRAK protein in the presence of ERK3 (Fig 4B, asterisk). As previously demonstrated, ERK4 and MK5 interact and mutually phosphorylate each other. The increased phosphorylation of ERK4 by MK5 is easily detectable by a decreased mobility of ERK4 in SDS-gel electrophoresis [23]. We also used this assay to monitor the catalytic activity of MK5/PRAK here. Again, expression of wild type MK5/PRAK protein resulted in the mobility shift of ERK4, while overexpression of inactive MK5/PRAK-T182A (as negative control) and of both deletion mutants does not change electrophoretic mobility of ERK4, which would point towards an absence of catalytic activity of MK5/PRAK-ΔEx6 and MK5/PRAK-ΔEx8 against ERK4 (Fig 4A and S6 Fig). However, we can not completely rule out catalytic activity of the mutants against other substrates, especially not for MK5/PRAK-ΔEx8, since this deletion mutant carries all subdomains (I to VII) required for catalytic activity [24]. In this regard it is interesting that we were able to detect auto-phosphorylation of MK5/PRAK and MK5/PRAK-ΔEx8, but not MK5/PRAK-ΔEx6, in the absence of GST-p38 in an *in vitro* kinase assay (Fig 5C, asterisk) indicating some constitutive kinase activity of WT and MK5/PRAK-ΔEx8 only.

## Discussion

For the generation of mouse null mutants the insertion of a drug resistance marker into an exon critical for gene function is a frequently used strategy. However, this strategy bears the risk of the formation of C-terminal truncated proteins capable of interfering with the targeted genes’ function. More importantly, skipping of the mutated exons due to aberrant splicing may lead to the expression of targeted proteins lacking a specific exon (reviewed in [25]). Exon skipping was observed in early studies of targeted deletions of DNA methyltransferase [26] or of the cell adhesion molecule L1 (Dahme, 1997). Here, we demonstrate that exon skipping proceeds as a result of targeting exon 6 or 8 of the protein kinase MK5/PRAK, resulting in detectable levels of the corresponding MK5/PRAK mRNAs and truncated proteins. An assignment of the kinase subdomains to the different exons of MK5/PRAK (Fig 2A) revealed that exon 6 codes for kinase subdomains VIa and VIb, which are necessary for catalytic activity [27]. Exon 8 does not code for a conserved protein kinase subdomain and its deletion would still allow the existence of a remnant mRNA coding for a protein kinase carrying the essential subdomains I-VIII (cf. Fig 2A). Although we were unable to detect any catalytic activity of the MK5/PRAK mutants against Hsp25/27 and ERK4 in our studies, we cannot exclude some cryptic catalytic activity of the MK5/PRAK-Δex8 protein towards other substrates. In line with this possibility is the observed auto-phosphorylation of GST-MK5/PRAK-Δex8 in *in vitro* kinase assays. A similar situation was recently described for secreted mammalian protein kinases of the Fam20 family [24], which display catalytic activity against casein and further extracellular glycoproteins, but carry only the conserved kinase subdomains I to VII.

Significant differences between the exon-deletion mutants of MK5/PRAK were detected with regard to their stability and subcellular localization (Figs 3 and 4). Different stability of the mutant proteins can be due to folding defects and/or differences in recognition by stabilizing or destabilizing binding partners. While wild type MK5/PRAK and Δex8 are located in the nucleus, Δex6 is artificially located in the cytoplasm. It is known that subcellular localization could strongly influence the stability of a specific protein by its ability to be ubiquitinated by compartment-specific E3 ligases and by the existence of compartment-specific pathways to proteasomal degradation. Furthermore, compartment-specific interaction partners can stabilize or destabilize the protein. Hence, we speculate that the artificial localization of the Δex6 mutant could be the reason for its instability.

Altered recognition by binding partners or intramolecular interactions could also account for changes in subcellular localization, while the NLS and NES *per se* are not affected in the MK5/PRAK mutants. For the related protein kinase MK2, a regulatory intramolecular interaction between the catalytic core and the C-terminus carrying NES/NLS have been described [28,29]. It could well be that the functions of the NLS and/or NES in the C-terminus of MK5/PRAK (both coded by exon 11) are also modulated by its intermolecular interaction with parts of the catalytic core (coded by exons 2–10). Interestingly, Dingar et al. [30] demonstrated that splice variants of MK5/PRAK lacking exons 2–6 were located in the cytoplasm, while the other variants were nuclear. This strongly suggests that such intermolecular interaction exists between the C-terminus and the small N-terminal lobe of the catalytic core of MK5/PRAK. This hypothesis is supported by the notion that co-expression of the MK5/PRAK interaction partner ERK3 (which prevents the intramolecular interaction by binding to the C-terminus of MK5/PRAK [31]), also leads to cytoplasmic localization of WT MK5/PRAK and Δex8 (Fig 4). Since subcellular localization of the mutants could only be analyzed for exogenously expressed tagged proteins, it still remains open whether the endogenous remnant proteins show the absolute identical localization.

Importantly, while stability and nuclear localization of wild type MK5/PRAK and -Δex8 are similar, MK5/PRAK-Δex6 displays instability and cytoplasmic localization. Hence, it appears unlikely that MK5/PRAK-Δex6 could overtake the function of wild type MK5/PRAK. However, because of similar stability and identical nuclear localization, MK5/PRAK-Δex8 might well be able to interfere with some function of the wild type protein in heterozygous and knockout cells. Thus increased skin tumor formation [6] and the enhanced hematopoietic tumorigenesis in the Δex8 strain [12] as well as the enhanced Ras-induced transformation of Δex8 MEFs [6] might be due to a pro-oncogenic effect of the MK5/PRAK-Δex8 mutant protein rather than a result of lacking the wild type kinase and its postulated tumor suppressive properties. Furthermore, pro-oncogenic properties of the MK5/PRAK-Δex8 protein would also explain the gene dosage effect observed in the skin carcinogenesis model using the Δex8 mice, where +/Δex8 mice had a tumor free survival rate which was clearly intermediate between the survival rates of Δex8/Δex8 and +/+ mice [6]. Hence, the controversial phenotypes of the two analyzed MK5/PRAK knockout models, regardless whether they are based on the biochemical properties of the remaining mutant proteins described here or on other non-identified differences between the mouse strains, challenge the tumor suppressive role ascribed to this kinase by the analysis of the MK5/PRAK-Δex8 strain.

## Materials and Methods

### One-step skin carcinogenesis model

Postnatal day 1–5 MK5/PRAK +/+, MK5/PRAK +/Δex6, or MK5 Δex6/Δex6 littermates (129 x C57BL/6) were treated with a single application of 50 μl 0.5% DMBA diluted in acetone to the dorsal surface. The mice were then monitored daily by staff personal at the Animal Facility of Aarhus University supervised by chief veterinarian Frederik Dagnæs for significant disadvantages resulting from the experiment. Tumor formation was analyzed and recorded weekly over a period of 50 weeks. In the case that the mice behaved in a manner, which suggested significant disadvantage as a result of the experiment (for example: abnormal movement pattern around the cages, crouching attitude, lack of interest in food and water, lethargy or other symptoms suggesting compromised health), the animals were sacrificed by cervical dislocation. There was no need for analgesics or anesthetics in this experiment. The work was approved by the Danish Laboratory Animal Research Committee. Mice were housed in specific pathogen-free conditions in accordance with European Union regulations.

### Antibodies and reagents

Antibodies against Ras, ERK3, p53, phospho-p53 (S15 (9284), S37 (9289) and S46 (2521)) and phospho-Hsp27 (Ser82) were obtained from Cell Signaling Technology, anti-PRAK antibody from Sigma and anti-GAPDH (glyceraldehyde-3-phosphate dehydrogenase) antibody from Millipore. Anti-GFP (B2), anti-GST (B-14), anti-EF-2 (C-14), Hsp27 (C-20) and secondary antibodies were purchased from Santa Cruz Biotechnology. Control siRNA (Allstars negative control), HiPerFect transfection and TopTaq DNA polymerase reagent were obtained from Qiagen, mouse MK5/PRAK siRNA (sc-36311) from Santa Cruz Biotechnology, [γ-^33^P]ATP from Hartmann Analytic, LMP Agarose from Bethesda Research Laboratories and SensiFAST SYBR No-Rox Kit and Vybrant DyeCycle Ruby Stain from Bioline.

### Plasmids and cloning

GFP-MK5 (pEGFP-C1-MK5), GST-MK5 (pGEX-MK5), GST-ERK3 (pDEST27-ERK3), His-ERK3 (pDEST26-ERK3), GST-ERK4 (pDEST27-ERK4), GST-Hsp27 (pDEST27-Hsp27), and GST-p38 (pDEST27-p38alpha) constructs were described earlier (Schumacher et al., 2004; Kant et al., 2006; Ronkina et al., 2007). MK5/PRAK-Δex6 deletion mutants were generated by PCR mutagenesis with the primer combinations: 5’-GACGCCCCTGTGAAATTATGTG-3’(forward)/5’-CTGCTTTGTTACTTGGCTGGC-3’(reverse) and MK5/PRAK-Δex8 deletion mutants were generated by PCR amplification with the primer combinations: 5’-AGCTGTGACTTGTGGTCCCTAG-3’(forward)/5’-CTGAGGTGCTACATAGTAAGGG-3’(reverse). For GFP-tagged mutants pEGFP-C1-MK5 construct was used as a template and for GST-tagged mutants pGEX-MK5 was used.

### Cell culture

WT and Δex6 primary MEFs were generated and maintained as reported previously [9]. Δex8-primary MEFs were kindly provided by Drs. Sun and Han. MEFs were immortalized by SV40-largeT as described earlier (Ronkina et al., 2007).

### Detection of Subcellular Localization of GFP-tagged Proteins

HEK293 cells were transiently transfected by polyethylenimine reagent in Chamber slides (Nunc Lab-Tek). Imaging was performed 24 h posttransfection using a Leica TCS SP2 confocal microscope with standard settings. For nuclear staining Vybrant DyeCycle Ruby Stain was added to CO_2_-independent medium to the final concentration of 5μM 30min before imaging.

### GFP pull-down and kinase assays

HEK-293T cells were transfected in 10 cm plates with the indicated expression constructs and at 24 h post-transfection cells were washed once with PBS and lysed in 200μl lysis buffer (10mM Tris/HCl (pH 7.5); 150mMNaCl; 0.5mM EDTA; 0.5% NP-40; 0.2mM PMSF, 1mM benzamidin, 1μg/ml Pepstatin A, 0.1% b-ME). After 30min incubation on ice lysates were clarified by centrifugation for 20min at 4C and 600μl of dilution buffer (10mM Tris/HCl (pH 7.5); 150mM NaCl; 0.5mM EDTA; 0.2mM PMSF, 1mM benzamidin, 1μg/ml Pepstatin A, 0.1% b-ME) was added. The resulting lysates were incubated with GFP nanobodies [32] coupled to magnetic epoxy beads (M-270 Dynal/Life Technologies) for 1h at 4°C. The nanobody was purified as described in [32] and the coding plasmid was a kind gift from Dr. Andrzej Dziembowski (Warsaw, PL). After precipitation samples were washed two times with dilution buffer, then two times with wash buffer (10mM Tris/HCl (pH 7.5); 300mMNaCl; 0.5mM EDTA) and finally once with glycerophosphate buffer (50mM Na-b-glycerophosphate; 0.1mM EDTA, pH7.4). Beads were re-suspended in 20μl of kinase buffer (50mM Na-b-glycerophosphate pH7.4; 0.1mM EDTA, 10μg recombinant Hsp27) and 5 μl of hot ATP mixture (20 mM MgCl2, 0.5 mM ATP, 0.1 μl of [γ-^33^P]-ATP) were added to the reaction mix. Kinase reaction was carried out at 30°C for 1 h, was stopped by adding 8 μl of 4x Laemmli buffer and samples were heated to 96°C for 5 min. Protein samples were separated on a 7.5–22.5% polyacrylamide gradient gel. After separation gel was used for Western blotting as described earlier [33] or protein were fixed in the gel using a coomassie staining-solution (250 mg/L Coomassie G, 40% methanol, 10% glacial acetic acid). After destaining the gel was placed on filter paper and dried for 2 h at 70°C using a gel dryer. Radioactivity incorporated into substrates was quantified by phosphoimaging using the FLA9000 imager (Fuji).

### Protein purification and *in vitro* kinase assay

GST-tagged MK5/PRAK-WT (wild-type) and -Δex6 or -Δex8 mutants were expressed in Escherichia coli BL21 and solubilized by sonication. Proteins were purified using Protino Glutathione Agarose 4B (Machery & Nagel), eluted with elution buffer (10mM glutathione; 50mM Tris-HCl pH 8.0; 1% Triton X-100) and dialyzed against dialysis buffer (50mM Tris-HCl pH8.0; 150mM NaCl; 50% glycerin; 0,5mM DTT, 1mM benzamidin, 0,2mM PMSF; 0,1% b-ME). 3 μg of recombinant GST-tagged MK5/PRAK-WT (wild-type) or -Δex6 or -Δex8 mutant protein were taken for each kinase reaction alone or in combination with 3 μg of recombinant GST-p38. Kinase reaction was carried out as described above.

### Luciferase reporter assay

MCF7 cells were co-transfected using Lipofectamine 2000 reagent with 100 ng of PG13-luciferase reporter [34], 100 ng of promoter-less pGL4.70 renilla construct and indicated amounts of pEGFP-MK5 or pDEST27-p38. The total amount of DNA per well was kept constant (0.6μg per well in 24-well plate) by adding GST or GFP expression vectors. Luciferase activity was measured 26 hr after transfection and was normalized using the renilla signal.

### Primers and primer combinations for genotyping and mRNA detection

DNA was prepared from WT, Δex6 and Δex8 MEFs. Genotyping was performed by PCR analysis. Primers MK5-1833 (5’-CGTAACACTAGCCACAGTTGTAACTGA-3’) and TMK5 rc1 (5’-GCTTGAGGTCTCTGTGCGCAATG-3’) were used to detect the wild type allele with a PCR product of 168bp. Primers MK5-1833 (5’-CGTAACACTAGCCACAGTTGTAACTGA-3’) and neorc 120 (5’-GTTCATTCAGGGCACCGGACAGGTCG-3’) were used to detect Δex6 knockout allele with a PCR product of ~600bp. The other primers combination to detect wild type and Δex8 knockout allele were described in [6].

To detect MK5/PRAK mRNA, total RNA was isolated from WT, Δex6 and Δex8 MEFs using the NucleoSpin RNA purification method (Machery-Nagel) followed by reverse transcription (Fermentas). The follow primer combinations were used Exon1-forward (5’-AGCGACATGGAGAAAGCCAT-3’)/Exon5-reverse (5’- CTGCTTTGTTACTTGGCTGGC-3’) to amplify 453bp MK5/PRAK mRNA fragment spanning exons 1–5; Exon1-forward (5’-AGCGACATGGAGAAAGCCAT-3’)/Exon7-reverse (5’- CTGAGGTGCTACATAGTAAGGG -3’) to amplify 567bp MK5/PRAK mRNA fragment spanning exons 1–7 from WT and Δex8 MEFs or 477bp fragment lacking the 90 bases of exon 6 from Δex6 MEFs; Exon9-forward (5’- AGCTGTGACTTGTGGTCCCTAG -3’)/Exon11-revese (5’- CCACCGCCTTATCCATCATCA -3’) to amplify 316bp MK5/PRAK mRNA fragment spanning exons 9–11. Exon7-forward (5’- GACGCCCCTGTGAAATTATGTG-3’)/Exon11-revese (5’- CCACCGCCTTATCCATCATCA -3’) to amplify 493bp MK5/PRAK mRNA fragment spanning exons 7–11 from WT and Δex6 MEFs or 412bp fragment lacking the 81 bases of exon 8 from Δex8 MEFs.

PCR program: 94C 3min; 35 cycles: 94C 30sec, 60C 30sec, 72C 1min; 72C 10min.

Quantification of p21WAF mRNA was performed by RT-PCR using the primers p21mouse-forward: 5’-AAG TGT GCC GTT GTC TCT TC-3’ and p21mouse-reverse: 5’-ACT TCA GGG TTT TCT CTT GC-3’. Real-time PCR (Q-PCR) was carried out using SYBR Green chemistry for p21 transcript and Taqman assay (Applied Biosystems) for quantifying actin expression. PCR was performed on a Rotorgene 2000 real-time PCR instrument. The threshold cycle (CT) for each individual PCR products was calculated by the instrument software, and CT values obtained were normalized against actin mRNA.

### Anchorage-independent growth in soft agar

The assay was performed by following the standard procedure. Briefly, primary murine fibroblasts at passage 1–2 were transduced with H-Ras-V12 oncogene (pBabe-Puro-H-RasV12) or with empty vector (pBabe-Puro) as described earlier [8,35]. After 5 days of selection with 2μg/ml puromycin cells were collected and 3x10^4^ cells were re-suspended in a medium containing 0.3% low-melting-point agarose and plated onto a solidified bottom layer medium containing 0.5% agarose in 6 well plates. Medium was added to the top layer to prevent drying of the agarose gel. Colonies growth was monitored for up to 4 weeks and pictures of representative fields were taken using a Leica EC3 camera. Ras-expression was monitored by Western blot using Ras-antibodies and GAPDH as a control. p53-/- MEFs were derived from B6.129S2-Trp53 ^tm1 Tyj^ mice [36] kindly provided by Dr. K. L. Rudolph (Hannover and Ulm, Germany).

### Small RNA Interference (siRNA)

WT, Δex6 and Δex8 MEFs were transiently transfected with either control (Allstars negative control, Qiagen) or mouse MK5/PRAK siRNA (sc-36311) using the HiPerFect reagent (Qiagen) according to the manufacturer’s instructions. In brief, 4x10^4^ cells were seeded per well in 12-well plate. On the next day 150ng siRNA was diluted in 100 μl of culture medium without serum and antibiotics, followed by addition of 6μl of HiPerFect transfection reaction per one well. After incubation for 10min, the transfection complex was added to the cells covered with 1100μl complete medium. After 80hr of incubation cells were lysed in 200μl of 1x Laemmli buffer and 50μl lysates were applied for WB.

## Supporting Information

S1 Fig Comparison of tumor free survival of WT, MK5/PRAK-Δex6 and MK5/PRAK-Δex8 mice in the one-step DMBA skin tumor model. The graphs are derived from the measured values of Δex6 mice and the estimated and compiled ex8+/+ and Δex8 data from the graph of Fig 1A of [6,9]. WT1 designates the WT control of the Δex6 experiment while WT2 is the control of the experiment of [6,9]. There is no significant difference between the WT controls (log-rank test p = 0.372).
(TIF)

S2 Fig Phosphorylation of p53 at S15, S37 and S46 and transcriptional activation of p53 in MCF-7 cells does not depend on p38 and MK5/PRAK. Panel A) MCF-7 cells were stimulated by doxorubicin (1μM)- or UV (40J/m^2^)-treatment in the absence and presence of the p38 MAPK inhibitor BIRB796 (1μM) and phosphorylation of p53 at S15, 37 and 46 was monitored by site-specific antibodies (cell signaling technology CST #9284, 9289, and 2521, respectively). Although MK5/PRAK is significantly expressed, no p38/MK5-dependent phosphorylation of p53 could be detected. As positive control for p38 inhibition the phosphorylation of Hsp27 by the p38/MK2 pathway was monitored (pHsp27-S82). Panel B) Luciferase reporter gene assay for p53 activity in MCF-7 cells using PG13-Luc, which contains 13 copies of the p53-binding consensus sequence in front of the luc-reporter [34]. Co-transfection of p53 increases transcriptional activity in this assay, but this activity is independent of p38 MAPK and of catalytic activity of MK5/PRAK. The mutant MK5-T182A used cannot be phosphorylated and activated by p38 MAPK [18]. The amount of DNA transfected is indicated. Panel C) Peptide substrate array analysis of MK5 was performed as described in [39]. GST-MK5 activity was assayed by radiolabel incorporation from [γ-^33^P]ATP on a set of peptides with the general sequence Y-A-X-X-X-X-X-S/T-X-X-X-X-A-G-K-K(biotin) having the indicated amino acid present fixed at the indicated position relative to the phosphorylation site. The substrate consensus motif is compared to the sequence surrounding S37 in p53 of human and mouse. The prominent basic residue (R) in minus-3 position is missing in both p53 sequences.
(TIFF)

S3 Fig PCR genotyping results of WT, Δex6 or Δex8 primary MEFs distinguishing MK5/PRAK wild type (WT), Δex6 and Δex8 knockout alleles. Four different primer combinations were used: the upper panel represents the results of PCR where the same primer combinations were used as described in [6] to discriminate between MK5/PRAK WT and exon8 knockout genome, the lower panel represents the results of PCR with the primer combinations described in materials and methods to discriminate between MK5/PRAK WT and exon6 knockout genome.
(TIF)

S4 Fig Quantitative analysis of subcellular localization of GFP-MK5 and its truncated variants. A) Representative fluorescence microscopic images (magnification 200x) of subcellular localization of GFP and recombinant GFP-MK5/PRAK fusion proteins transfected into HEK293 cells used for the quantification in B). A nuclear co-staining in the living cells was performed using Vybrant DyeCycle Ruby Stain (life technologies). B) Quantification of subcellular localization of GFP and the GFP-fusion proteins. From three independent transient transfections more than 100 cells of each transfection were counted. Cells were scored predominantly nuclear (N), predominantly cytoplasmic (C) or equally distributed between nucleus and cytoplasm (NC). The cells with very strong and apparently toxic overexpression resulting in NC were not excluded here. We normally avoid such cells in quantification of transient transfections with GFP fusion proteins, but here the fully unbiased counting is presented.
(TIF)

S5 Fig GFP-MK5/PRAK and its exon6 and exon8 deletion mutants interact with His-ERK3. His-ERK3 was detected in immunoprecipitates using GFP-nanobodies and in whole lysate (as control) by Western blot against ERK3. Western blot against GFP was used to detect GFP-MK5/PRAK and its mutant variants.
(TIF)

S6 Fig Exon 6 (Δex6) and Exon 8 (Δex8) deletion mutants of MK5/PRAK do not phosphorylate ERK4. HEK293 cells were transfected with indicated constructs. 24 hr post-transfection cells were lysed in the plates and analyzed by Western blotting using the indicated antibodies. A slower migrating band of GST-ERK4, indicating its phosphorylation, appears when co-expressed with GFP-MK5/PRAK wild type, but not with GFP-MK5/PRAK-Δex6, GFP-MK5/PRAK-Δex8 or kinase-dead GFP-MK5/PRAK-T182A mutant.
(TIF)
