# Supplementary figures and images for: Comparative Analysis of Two Gene-Targeting Approaches Challenges the Tumor-Suppressive Role of the Protein Kinase MK5/PRAK
Source: PLoS One. 2015 Aug 21;10(8):e0136138. doi: 10.1371/journal.pone.0136138 (PMC4546416; doi:10.1371/journal.pone.0136138)

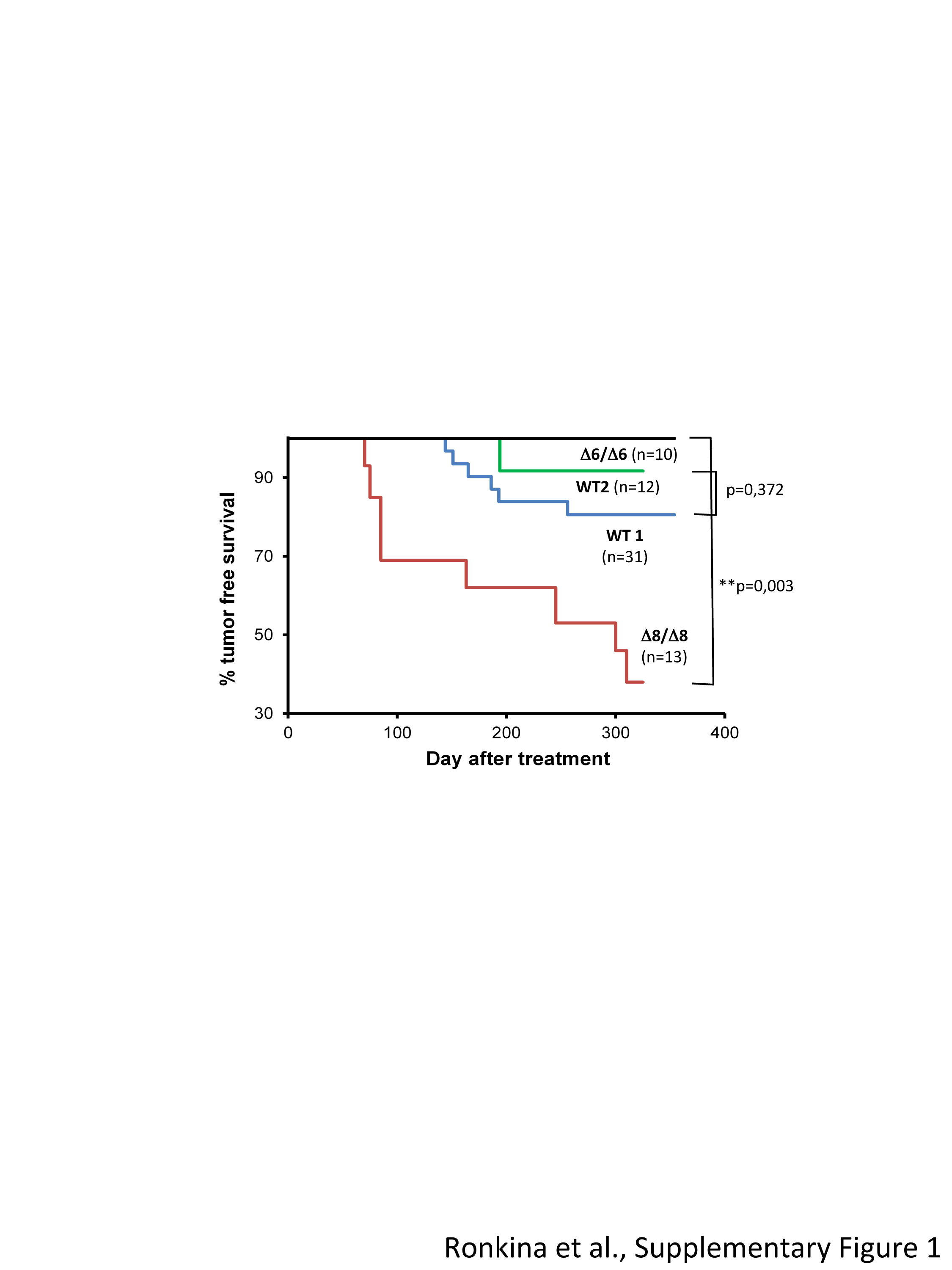

Supplement: S1 Fig — The graphs are derived from the measured values of Δex6 mice and the estimated and compiled ex8+/+ and Δex8 data from the graph of Fig 1A of [6,9]. WT1 designates the WT control of the Δex6 experiment while WT2 is the control of the experiment of [6,9]. There is no significant difference between the WT controls (log-rank test p = 0.372). (TIF) [file pone.0136138.s001.tif]

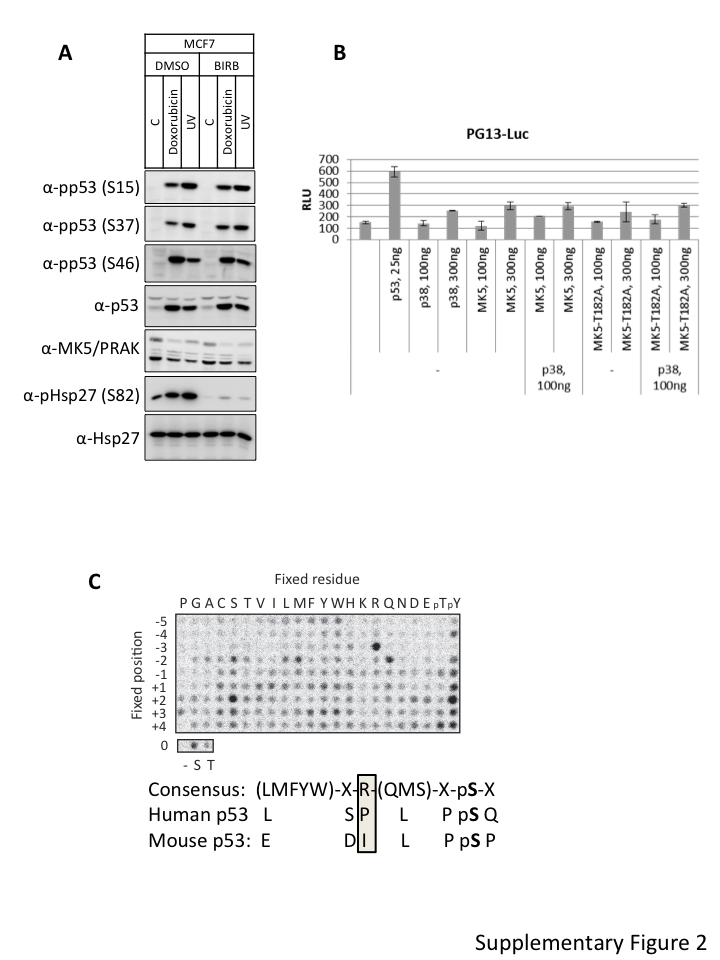

Supplement: S2 Fig — Panel A) MCF-7 cells were stimulated by doxorubicin (1μM)- or UV (40J/m2)-treatment in the absence and presence of the p38 MAPK inhibitor BIRB796 (1μM) and phosphorylation of p53 at S15, 37 and 46 was monitored by site-specific antibodies (cell signaling technology CST #9284, 9289, and 2521, respectively). Although MK5/PRAK is significantly expressed, no p38/MK5-dependent phosphorylation of p53 could be detected. As positive control for p38 inhibition the phosphorylation of Hsp27 by the p38/MK2 pathway was monitored (pHsp27-S82). Panel B) Luciferase reporter gene assay for p53 activity in MCF-7 cells using PG13-Luc, which contains 13 copies of the p53-binding consensus sequence in front of the luc-reporter [34]. Co-transfection of p53 increases transcriptional activity in this assay, but this activity is independent of p38 MAPK and of catalytic activity of MK5/PRAK. The mutant MK5-T182A used cannot be phosphorylated and activated by p38 MAPK [18]. The amount of DNA transfected is indicated. Panel C) Peptide substrate array analysis of MK5 was performed as described in [39]. GST-MK5 activity was assayed by radiolabel incorporation from [γ-33P]ATP on a set of peptides with the general sequence Y-A-X-X-X-X-X-S/T-X-X-X-X-A-G-K-K(biotin) having the indicated amino acid present fixed at the indicated position relative to the phosphorylation site. The substrate consensus motif is compared to the sequence surrounding S37 in p53 of human and mouse. The prominent basic residue (R) in minus-3 position is missing in both p53 sequences. (TIFF) [file pone.0136138.s002.tiff]

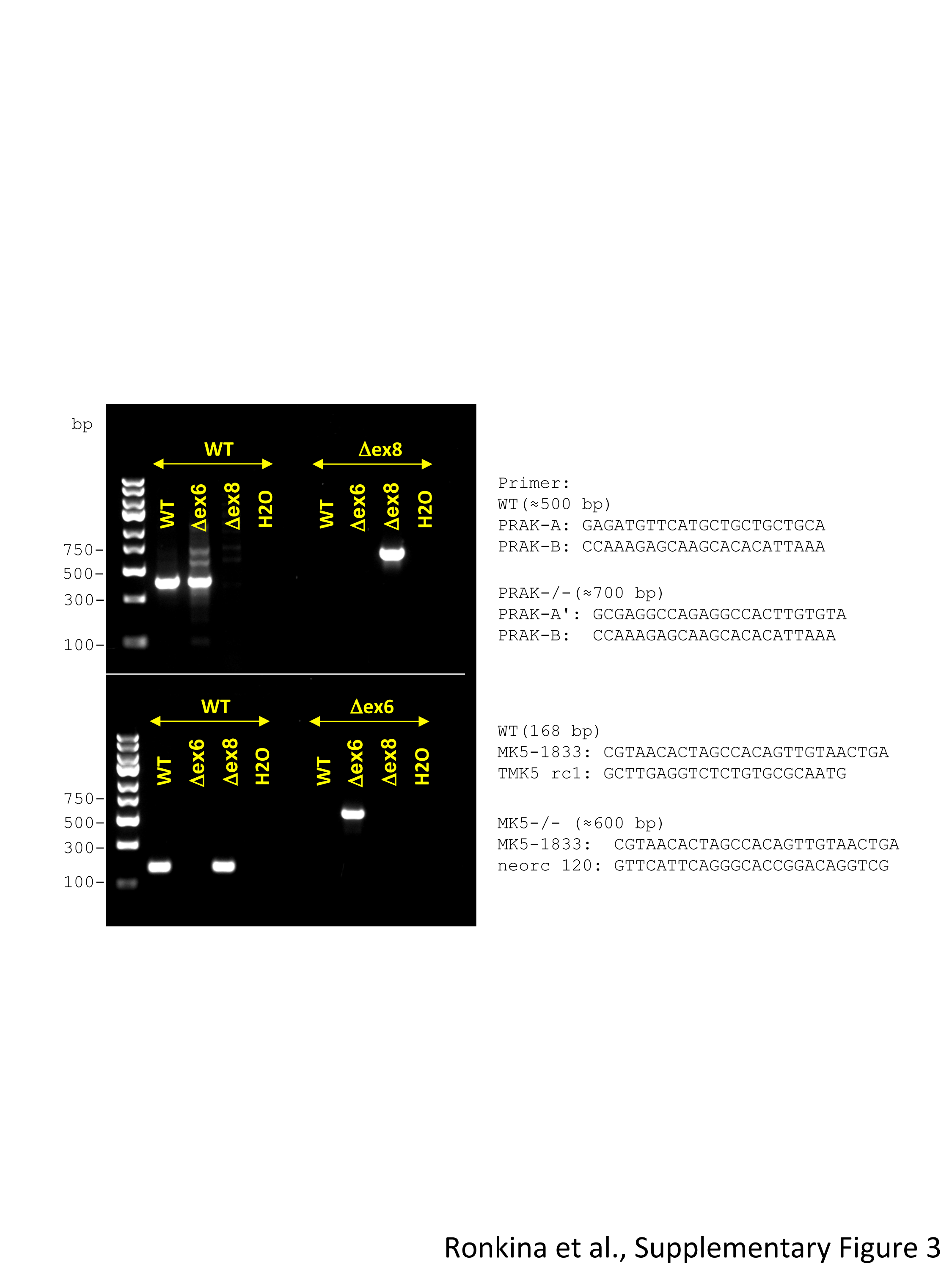

Supplement: S3 Fig — Four different primer combinations were used: the upper panel represents the results of PCR where the same primer combinations were used as described in [6] to discriminate between MK5/PRAK WT and exon8 knockout genome, the lower panel represents the results of PCR with the primer combinations described in materials and methods to discriminate between MK5/PRAK WT and exon6 knockout genome. (TIF) [file pone.0136138.s003.tif]

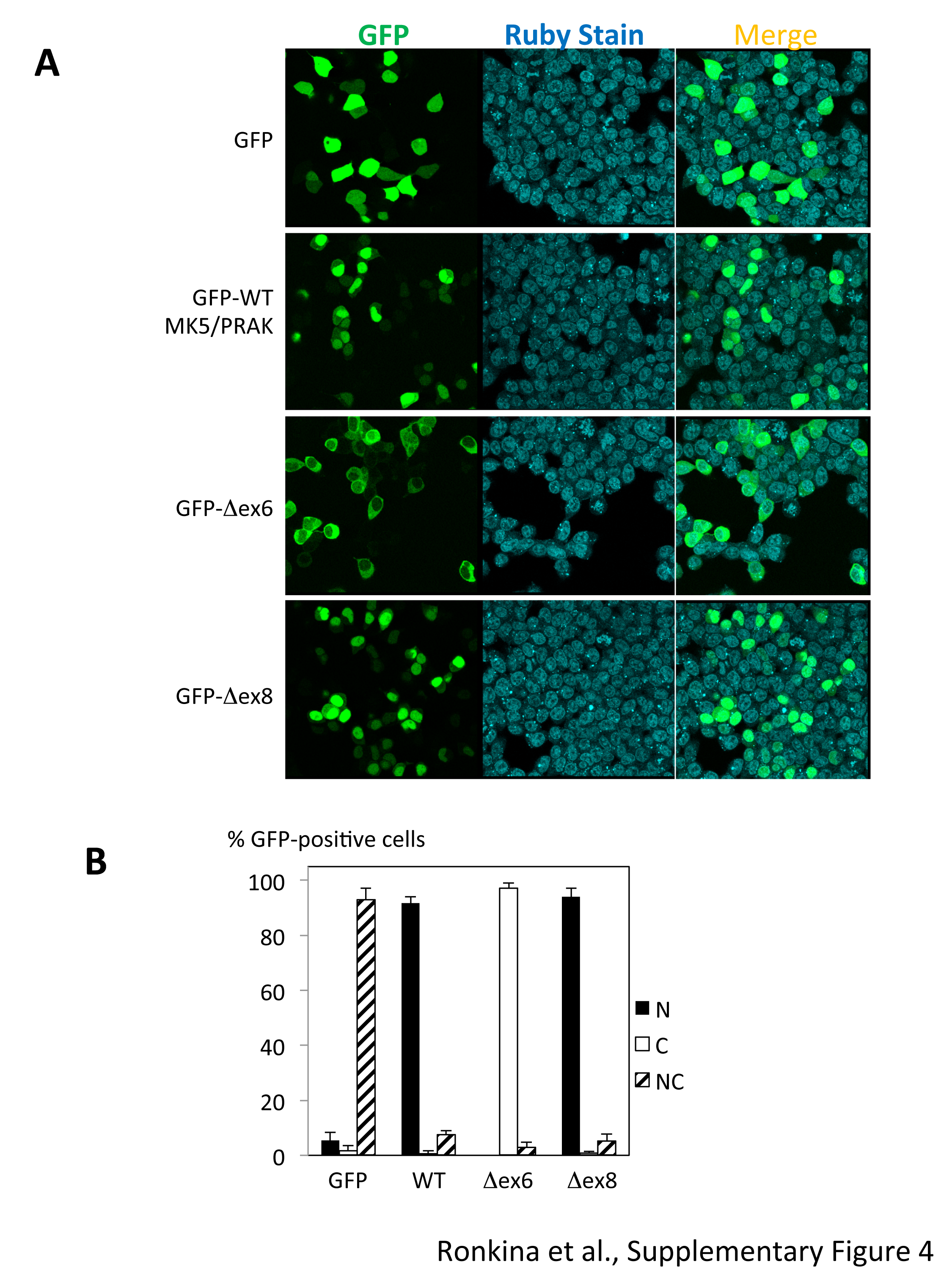

Supplement: S4 Fig — A) Representative fluorescence microscopic images (magnification 200x) of subcellular localization of GFP and recombinant GFP-MK5/PRAK fusion proteins transfected into HEK293 cells used for the quantification in B). A nuclear co-staining in the living cells was performed using Vybrant DyeCycle Ruby Stain (life technologies). B) Quantification of subcellular localization of GFP and the GFP-fusion proteins. From three independent transient transfections more than 100 cells of each transfection were counted. Cells were scored predominantly nuclear (N), predominantly cytoplasmic (C) or equally distributed between nucleus and cytoplasm (NC). The cells with very strong and apparently toxic overexpression resulting in NC were not excluded here. We normally avoid such cells in quantification of transient transfections with GFP fusion proteins, but here the fully unbiased counting is presented. (TIF) [file pone.0136138.s004.tif]

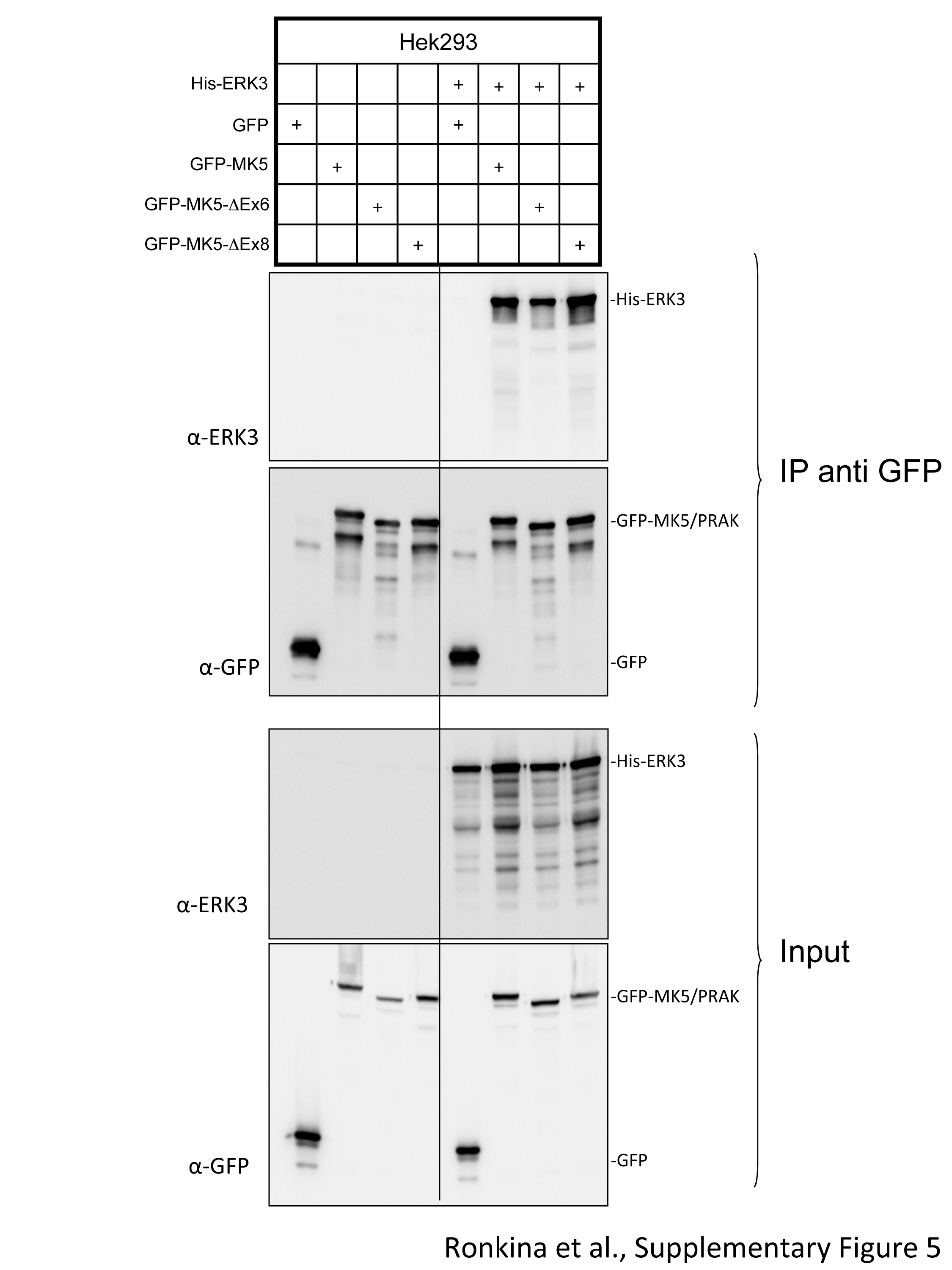

Supplement: S5 Fig — His-ERK3 was detected in immunoprecipitates using GFP-nanobodies and in whole lysate (as control) by Western blot against ERK3. Western blot against GFP was used to detect GFP-MK5/PRAK and its mutant variants. (TIF) [file pone.0136138.s005.tif]

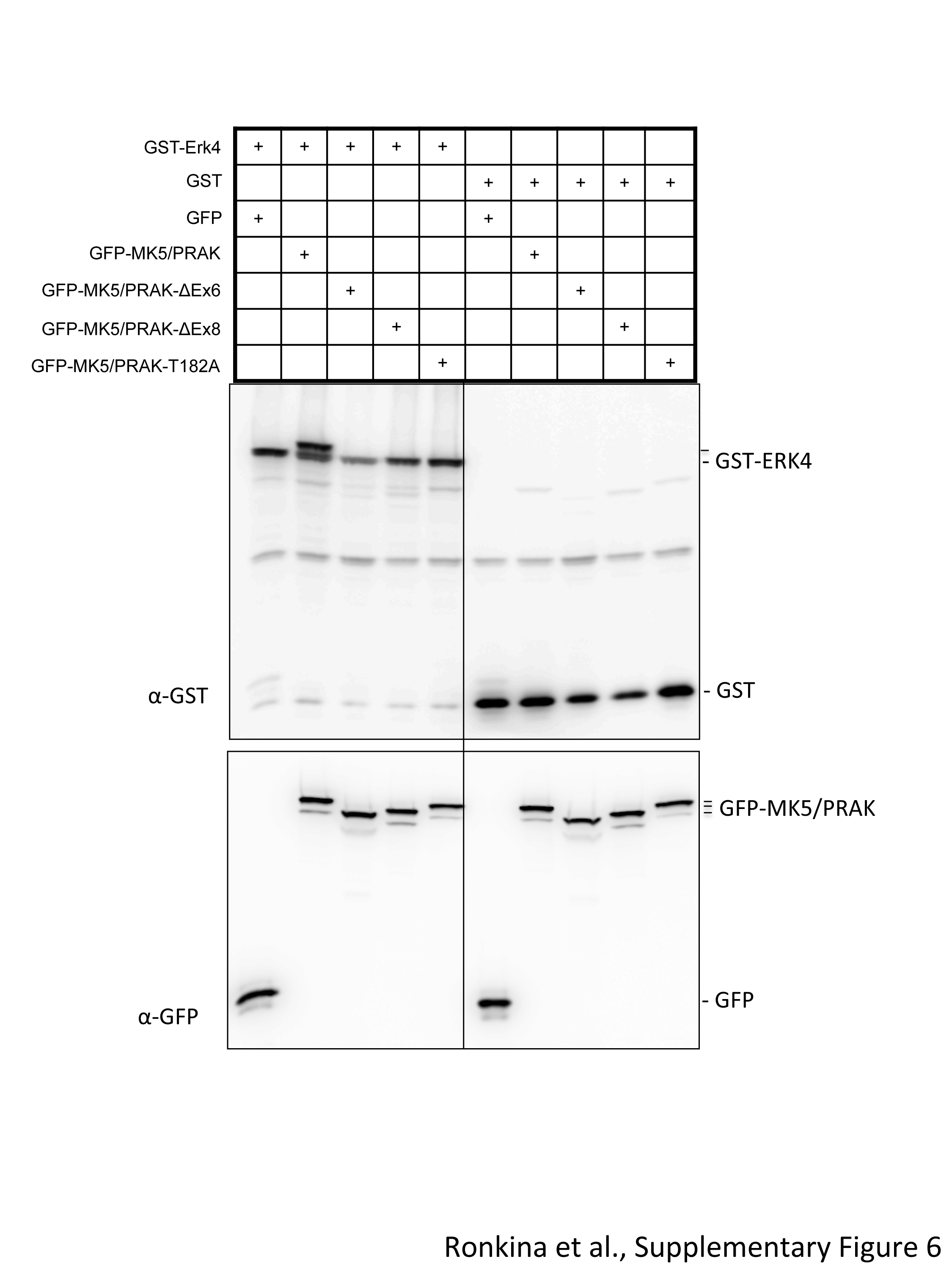

Supplement: S6 Fig — HEK293 cells were transfected with indicated constructs. 24 hr post-transfection cells were lysed in the plates and analyzed by Western blotting using the indicated antibodies. A slower migrating band of GST-ERK4, indicating its phosphorylation, appears when co-expressed with GFP-MK5/PRAK wild type, but not with GFP-MK5/PRAK-Δex6, GFP-MK5/PRAK-Δex8 or kinase-dead GFP-MK5/PRAK-T182A mutant. (TIF) [file pone.0136138.s006.tif]
